# Numerical Study on the Distribution of Rodlike Particles in Laminar Flows of Power Law Fluids Past a Cylinder

**DOI:** 10.3390/polym15081956

**Published:** 2023-04-20

**Authors:** Wenqian Lin, Zhenna Li, Shanliang Zhang, Jianzhong Lin

**Affiliations:** 1 School of Media and Design, Hangzhou Dianzi University, Hangzhou 310018, China; 2 State Key Laboratory of Fluid Power Transmission and Control, Zhejiang University, Hangzhou 310027, China

**Keywords:** rod-like particles, power-law fluid, spatial distribution, orientation, numerical simulation

## Abstract

The contraction/expansion laminar flow containing rodlike particles in power-law fluid is studied numerically when the particles are in a dilute phase. The fluid velocity vector and streamline of flow are given at the finite Reynolds number (Re) region. The effects of Re, power index *n* and particle aspect ratio *β* on the spatial and orientation distributions of particles are analyzed. The results showed that for the shear-thickening fluid, particles are dispersed in the whole area in the contraction flow, while more particles are gathered near the two walls in the expansion flow. The spatial distribution of particles with small *β* is more regular. *Β* has a significant, *n* has a moderate, but Re has a small impact on the spatial distribution of particles in the contraction and expansion flow. In the case of large Re, most particles are oriented in the flow direction. The particles near the wall show obvious orientation along the flow direction. In shear-thickening fluid, when the flow changes from contraction to expansion, the orientation distribution of particles becomes more dispersed; while in shear-thinning fluid, the opposite is true. More particles orient to the flow direction in expansion flow than that in contraction flow. The particles with a large *β* tend to align with the flow direction more obviously. Re, *n* and *β* have great influence on the orientation distribution of particles in the contraction and expansion flow. Whether the particles initially located at the inlet can bypass the cylinder depends on the transverse position and initial orientation of the particles at the inlet. The number of particles with *θ*_0_ = 90° bypassing the cylinder is the largest, followed by *θ*_0_ = 45° and *θ*_0_ = 0°. The conclusions obtained in this paper have reference value for practical engineering applications.

## 1. Introduction

The flow containing rodlike particles is very common in chemical industry, materials, environmental protection and other industries. There are several important factors that determine the characteristics of such flow, and the most important factors are the spatial distribution and orientation of rodlike particles in the fluid.

The motion of rodlike particles in flows is complicated because the particle rotation and orientation are coupled with the translation motion. The particles will exhibit nonuniform spatial distribution and non-isotropic orientation distribution due to the difference of the flow velocity and shear rate in different region, which has aroused people’s attention. Altan et al. [1] adopted the model of Dinh-Armstrog [2] and obtained the orientation distribution of particles by solving the equation of the direction tensor in a channel flow based on the assumption that the particles moved in a plane, and the rodlike particles are assumed to be rigid cylindrical bodies with negligible inertia. Chono and Makino [3] obtained the spatial and orientation distributions of particles by solving the equation of directional tensor combined with the equivalent strain tensor model and with considering the effect of particles on the fluid for the flow between two plates. Chiba and Nakamura [4] studied two-dimensional orientations of 1800 fibers in a Newtonian flow through a 1:4 backward-facing step channel, and found that the fibers with large aspect ratio (*β* = 10,000) are completely arranged along the streamline direction, while the fibers with small aspect ratio (*β* = 5) have a dominant orientation. Chiba et al. [5] solved the kinetic energy equation coupled with distribution function of fiber orientation for the flow between two plates, and the suspension consisted of high aspect-ratio 180 rigid fibers in a Newtonian fluid. They showed that the non-isotropic distribution of fiber orientation and stress occurred near the entrance, and the inlet region presented a significant non-Newtonian flow effect with the increase of fiber concentration and aspect ratio. Lin et al. [6] used three-dimensional vortex method to simulate the circular jet flow, and then calculated the fiber motion in flow by particle trajectory model without considering the effect of particle on the flow, finally obtained the spatial and orientation distributions of fibers under different Reynolds number, Stokes number and fiber aspect ratio. Lin et al. [7] compared various forces exerted on a moving rod-like particle in an incompressible elongational-shear flow, and indicated that the Stokes resistance, Basset force and added mass were much larger than the Magnus force, Saffman force and pressure gradient force.

Cai et al. [8] studied the gas-solid two-phase turbulent flow of rod-like particles with two-way coupling approach, and showed that the volume fraction of particles in central region was higher than that in near-wall region, the velocity and pressure of the flow decreased evidently in the region where the volume fraction of particles was high. They also obtained the distributions of pressure, velocity and turbulent kinetics along the axis of the riser [9]. Hao et al. [10] simulated numerically the frictional rod-like particle shear flows with different size distributions (monodisperse, binary, Gaussian, uniform), it was found that stress fluctuation reached the maximum, and stress rate increased with the increase of volume fraction at the jamming volume fraction. The jamming volume fraction presented obvious dependences on the fraction of longer particles and particle size in the case of polydisperse particles. They also indicated that no segregation appeared in the absence of gravity and interstitial fluid medium. The minimum stress difference due to the change in volume fraction of a particle species was observed at volume fraction of 0.2 [11].

The investigations as shown above are related to the case of rod-like particles flowing in the Newtonian fluids. However, in many industrial applications, the fluid containing rodlike particles is non-Newtonian fluid. The scenario is even more complex when rodlike particles are suspended in non-Newtonian fluids due to the fluids itself show the rheological properties, so the dynamics of rodlike particles in non-Newtonian fluids has been becoming the focus which people greatly concern for. Leal [12] studied the translation of a slender axisymmetric rodlike particle through a quiescent second-order fluid and rotation in a simple shear flow of the same material, and the results showed that freely translating particles with fore-aft symmetry presented a single stable orientation. The rheological property caused a particle drift through Jeffery orbit to equilibrium orbit at small and moderate shear rates. The rate of orbit drift could be employed to determine the second normal stress difference which also controlled the drifting rate. Brunn [13] studied the motion of rigid particles in a homogeneous shear flow of a viscoelastic fluid with a creeping-motion equation, and indicated that rod-like particles moved towards a vorticity alignment in the flow plane and tumble around the vorticity axis. Cohen et al. [14] studied the particle orientation in a dilute suspension of rod-like particles in a second-order fluid and found that most particles oriented close to the vorticity axis when the fluid elasticity was strong enough to restrain the rotational diffusion of the particles. Iso et al. [15,16] showed that a single rodlike particle rotated towards the vorticity axis in weakly elastic fluids (100 ppm polyacrylamide), but aligned rapidly along the flow direction in highly elastic fluids (1000 and 2000 ppm polyacrylamide). In intermediate elastic fluids (200 and 500 ppm polyacrylamide), the particle had an orientation in between the flow and the vorticity direction. Gunes et al. [17] studied the flow-induced orientation of spheroidal particles in viscoelastic fluids with a wide range of rotational Peclet and Weissenberg numbers, and indicated that particle changed orientation from a random state to spinning in Jeffery orbits with increasing shear rate. At higher elasticity, particles reoriented again to the flow direction with the exception in Boger fluids. With suitable flow histories bimodal flow-vorticity orientation distributions could be generated. De Borzacchiello et al. [18] proposed simplified modeling of short rodlike particles in second-order fluids from microscopic to macroscopic scales, which could be used in industrial simulation software. Phan-Thien’s group [19,20] extended the complete double layer boundary integral equation formulation for Stokes flows to viscoelastic fluids to solve the mobility problem for a particle in an unbounded body of fluid, and showed that viscoelastic stresses slowed down rotation of prolate spheroid in a shear flow of Oldroyd-B fluid at a relatively small Deborah number. Lin et al. [21] studied the dynamics of rod-like particles in the contraction flow of a second-order fluid when the particles were in a dilute phase, and showed that the spatial and orientation distributions of particles were dependent on the inertia, viscoelasticity and effect of confined wall. High shear rate of the fluid made the particles align with the flow direction. Particle spatial distribution became more non-uniform, andmore particles tended to align with theflowdirection with increasing Deborah number and contraction ratio. Particle aspect ratio had a weaker effect on the particle distribution than Deborah number, Stokes number and contraction ratio. Lin et al. [22] explored the effect of various factors on the orientation distributions of rod-like particlesin a mixing layer of an Oldroyd-B fluid in the range of Stokes number (St) from 0.005 to 1.0, Weissenberg number (Wi) from 5 to 15, particle aspect ratio (α) from 5 to 25, and indicated that the particles with a small St were distributed homogenously. More particles aligned on the flow-gradient plane with the increase of Wi, and the decrease of St and α. Wi had a stronger effect on the orientation distribution than St and α. Stover and Cohen [23] studied the motion of suspended rodlike particles in the flow between two flat plates of at low Reynolds numbers, and obtained the data for rodlike particles with aspect ratios of 12.0 suspended in a Newtonian fluid; and for rodlike particles with aspect ratios between 5 and 8 in a non-Newtonian fluid. The results showed that, for the Newtonian fluid, particles aligned with the flow direction and less than a particle half-length from a wall interacted irreversibly with the wall; for the non-Newtonian fluid, particles that were aligned with the flow direction and were near walls did not rotate. Domurath et al. [24] studied numerically the properties of power-law fluids filled with rigid rods of different aspect ratios, and found that there was no similarity between the rheological coefficients for these particles at large aspect ratios; there were negligible differences in the angular velocities between the Newtonian and power-law matrix fluids, especially for large aspect ratios.

As mentioned above, the dynamics of rodlike particles in some kind of non-Newtonian fluids were investigated, but the study in power-law fluid with shear-dependent viscosity is rare. Power-law fluid is very common, for example, low solid mud, polymer drilling fluid, high concentration starch paste, pulp and paint with high solid content. In addition, most of the flows mentioned above are unbounded flow or parallel flow. However, in practical applications, many cases are contraction and expansion flow [25,26], i.e., the flow first passes through a contraction channel and then enters an expansion channel. So far, the authors have not seen any research report on the contraction and expansion flow of power-law fluid containing rodlike particles, although this kind of flow has strong application background. Therefore, in this paper, a set of equations for the contraction and expansion flow of power-law fluid containing rodlike particles are established and solved by numerical simulation, aiming to illustrate the influence of Reynolds number, power-index and particle aspect ratio on the spatial and orientation distributions of particles.

## 2. Model and Equation

### 2.1. Particle and Flow Model

**Figure 1:**
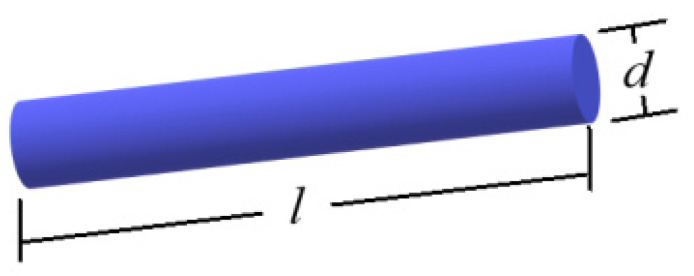
Schematic of a particle.

Figure 1 shows a rigid rodlike particle without Brown motion. The particle size is much smaller than the geometric size of the flow (the ratio of particle length to cylinder radius is *l*/*R* = 0.13). Let the particles be a dilute phase, ignoring the interaction between particles, and the effect of particles on the fluid. The effect of fluid on particles is reflected in the Stokes force caused by the velocity difference between the fluid and particles.

**Figure 2:**
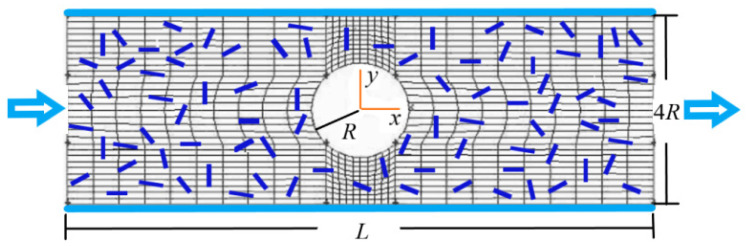
Schematic of a flow and grid division.

Figure 2 shows the flow and grid division. The power-law fluid containing rodlike particles enters from the left and flows out from the right. The upper and lower sides are solid walls, and there is a cylinder in the middle between the two walls.

### 2.2. Equation for Particle Motion

When particles move in the flow, they are affected by Stokes force, Basset force, additional mass force and oscillation, but the Stokes force ***F*** dominates in the case of present study by comparing the effects of these factors [7], and can be expressed as:

(1) F=μaD⋅U,

where *μ* is the fluid viscosity, *a* is the particle radius, ***U*** is the difference between the fluid and particles, ***D*** is the resistance coefficient and can be expressed as:

(2) $$D=\left[\begin{array}{cc} D_{//} & 0\\ 0 & D_{⊥} \end{array}\right],$$

where $D_{//}$ and $D_{⊥}$ are the resistance coefficients parallel to and perpendicular to the principal axis of particles, respectively, Loewenberg [27] presented the fitting curves of two resistance coefficients varying with the particle aspect ratio by experimental data:

(3) $$\left.\begin{array}{l} D_{//}=2.78715+\frac{27.2277}{\beta }-\frac{8.26673}{\beta^{2}}\\ D_{⊥}=4.78194+\frac{28.0349}{\beta }-\frac{10.0182}{\beta^{2}} \end{array}\right\}\beta \in (1,100),$$

where *β* is the particle aspect ratio. Dividing both sides of Equation (1) by the mass of particles *m_p_* = *ρ_p_πla*^2^, we have:

(4) $$\ddot{r}=\frac{C_{1}}{\mathrm{St}}A^{-1}DA\left(U_{f}-\dot{r}\right).$$

where $\ddot{r}$ is the particle acceleration, $\dot{r}$ is the particle velocity, ***U****_f_* is the fluid velocity at particle center, *C*_1_ = *U*/*πβL* with *L* and *U* being the characteristic length and velocity of the flow, respectively, ***A*** is transition matrix for changing particle from body coordinate to rectangular coordinate:

(5) $$A=\left[\begin{array}{cc} \cos\theta & \sin\theta \\ -\sin\theta & \cos\theta \end{array}\right],$$

where *θ* is the angle between particle principal axis and horizontal direction. St in Equation (4) is Stokes number:

(6) $$\mathrm{St}=\rho_{p}a^{2}U/\mu L,$$

where *ρ_p_* is the particle density.

The moment acting on particles can be obtained from the slender body theory [28]:

(7) $$M=\frac{8}{3}\pi \mu \sigma L^{3}(C-\dot{\theta })[1-\sigma (\ln2-1.8333)],$$

where $\sigma =\ln^{-1}(2\beta )$, $C=-\sin\theta \cos\theta \left(\frac{\partial u_{p}}{\partial x}-\frac{\partial v_{p}}{\partial y}\right)+\cos^{2}\theta \frac{\partial v_{p}}{\partial x}-\sin^{2}\theta \frac{\partial u_{p}}{\partial y}$ with *u_p_* and *v_p_* being the velocity component of particle along the *x* and *y* direction, respectively. Dividing both sides of Equation (7) by the rotational inertia of particles *J_p_* = *m_p_*(*a*^2^ + *l*^2^/6)/2, we have:

(8) $$\ddot{\theta }=4\left(C-\dot{\theta }\right)\sigma \left[1-\sigma \left(\ln2-1.8333\right)\right]/\left(\mathrm{St}\cdot \mathrm{fst}\right),$$

where $\dot{\theta }$ and $\ddot{\theta }$ are the angular velocity and angular acceleration of particles, respectively, fst is a parameter with time dimension:

(9) $$\mathrm{fst}=\frac{3L}{4U}(\frac{1}{\beta^{2}}+\frac{1}{6}).$$

When particle aspect ratio *β* is larger than 10, Equation (9) can be approximated as fst = *L*/8*U* and the rotational inertia of particles is *J_p_* = *m_p_l*^2^/12.

### 2.3. Equations for Fluid

Equation (4) contains the fluid velocity ***U****_f_*, so the fluid velocity should be first given by solving equations for fluid before solving the equations for particles.

The continuity and momentum equations of the fluid are:

(10) $$\nabla \cdot U_{f}=0,$$

(11) $$\rho \frac{DU_{f}}{Dt}=-\nabla p+\nabla \cdot \tau ,$$

where ***U****_f_* is the fluid velocity, *ρ* is the fluid density, *p* is the pressure, *τ* is the shear stress $\tau =\mu \dot{\gamma }$ with *μ* the dynamic viscosity and $\dot{\gamma }$ the rate of shearing tensor:

(12) $$\dot{\gamma }=\frac{1}{2}\left[\left(\nabla U_{f}\right)+{\left(\nabla U_{f}\right)}^{T}\right].$$

For the power-law fluid, the shear stress can be expressed as:

(13) $$\tau =m{\left|\dot{\gamma }\right|}^{n-1}\dot{\gamma },$$

then the effective viscosity is:

(14) $$\mu =m{\left|\dot{\gamma }\right|}^{n-1},$$

where *m* is the flow consistency coefficient; *n* is the power-law index; *n* < 1, *n* = 1 and *n* > 1 correspond to shear-thinning, Newtonian and shear-thickening fluids, respectively; $\left|\dot{\gamma }\right|$ is the local shear rate:

(15) $$\left|\dot{\gamma }\right|=\sqrt{2{\left(\frac{\partial u_{f}}{\partial x}\right)}^{2}+2{\left(\frac{\partial v_{f}}{\partial y}\right)}^{2}+{\left(\frac{\partial u_{f}}{\partial x}+\frac{\partial v_{f}}{\partial y}\right)}^{2}},$$

with *u_f_* and *v_f_* are the velocity component of fluid along the *x* and *y* direction, respectively.

## 3. Numerical Simulation

The one-way coupling method is used for numerical solution here, i.e., considering only the effect of fluid on particles and ignoring the effect of particles on fluid. First solving the velocity of the fluid through Equations (10)–(15), then calculating the force and moment exerted by the fluid on the particles through Equations (1)–(3) and (7), and finally obtaining the acceleration, angular acceleration, velocity, angular velocity, trajectory and orientation of the particles through solving Equations (4) and (8).

### 3.1. Distribution of Fluid Velocity

A finite volume method is used to solve Equations (10)–(15). The grid division of the flow is generated using Gambit 2.0 as shown in Figure 2 where quadrilateral grid is selected. Gambit 2.0 will automatically generate the grid in Figure 2 when the number of grids and grid shape are determined. The SIMPLE and power-law scheme are used to deal with the term of velocity–pressure coupling and the convection term. A staggered mesh system and an alternating direction implicit method are used to solve the discretized equations.

### 3.2. Boundary Conditions

The no-slip condition is used on the walls. The condition of fully developed flow is used at the outlet. In Figure 2 it is assumed that the inlet is sufficiently far from the cylinder in the middle between the two walls, the presence of the cylinder has no effect on the fluid velocity distribution at the inlet. Therefore, the velocity distribution in a channel flow is used as the boundary condition at the inlet.

By solving Equations (10)–(15) in a channel flow, the analytical solution of fluid velocity along the *x* direction at the inlet can be obtained:

(16) $$u_{f}=\frac{n}{n+1}{\left(\frac{\Delta p}{2mL}\right)}^{1/n}[{\left(2R\right)}^{\frac{n+1}{n}}-y^{\frac{n+1}{n}}],$$

where *n* is the power index of the fluid; Δ*p* is the pressure drop; *m* is the flow consistency coefficient; *L* is the channel length and 2*R* is the half width of channel as shown in Figure 2. The corresponding mean velocity is:

(17) $${\bar{u}}_{f}=\frac{n}{\left(3n+1\right)}{\left(\frac{\Delta p}{2mL}\right)}^{\frac{1}{n}}{\left(2R\right)}^{\frac{n+1}{n}}.$$

### 3.3. Velocity, Angular Velocity and Spatial Position of Particles

30 randomly oriented particles are initially evenly distributed along the y-direction at the inlet. Every other time step, 30 particles enter the flow, and these particles pass through the flow and then exit the flow from the outlet. After the particles entering the flow and exiting the flow have stabilized, the statistics of the particles in the computational domain are performed to provide the spatial and orientation distribution characteristics of the particles. Therefore, the number of particles in the computational domain is not constant, and its value depends on the relevant parameters of the flow (Reynolds number) and particles (aspect ratio and initial orientation). The acceleration and angular acceleration of particles are calculated with Equations (4) and (8), and then the particle velocity, angular velocity, position and orientation angle at the next moment are calculated with the following formula:

(18) $$\begin{array}{l} {\dot{x}}_{i+1}={\dot{x}}_{i}+{\ddot{x}}_{i}dt,\;\;\;\;\;\;{\dot{y}}_{i+1}={\dot{y}}_{i}+{\ddot{y}}_{i}dt,\\ x_{i+1}=\frac{x_{i}+\left({\dot{x}}_{i-1}+{\dot{x}}_{i+1}\right)dt}{2},\;y_{i+1}=\frac{y_{i}+\left({\dot{y}}_{i-1}+{\dot{y}}_{i+1}\right)dt}{2},\\ \theta_{i+1}={\dot{\theta }}_{i}+{\ddot{\theta }}_{i+1}dt,\;\theta_{i+1}=\frac{\theta_{i}+\left({\dot{\theta }}_{i-1}+{\dot{\theta }}_{i+1}\right)dt}{2}. \end{array}$$

Equation (18) is numerically solved by the fourth-order Runge-Kutta method. The orientation distribution of particles can be obtained by statistically averaging the orientation angle of particles.

## 4. Results and Discussion

### 4.1. Fluid Velocity Vector and Streamline of Flow

**Figure 3:**
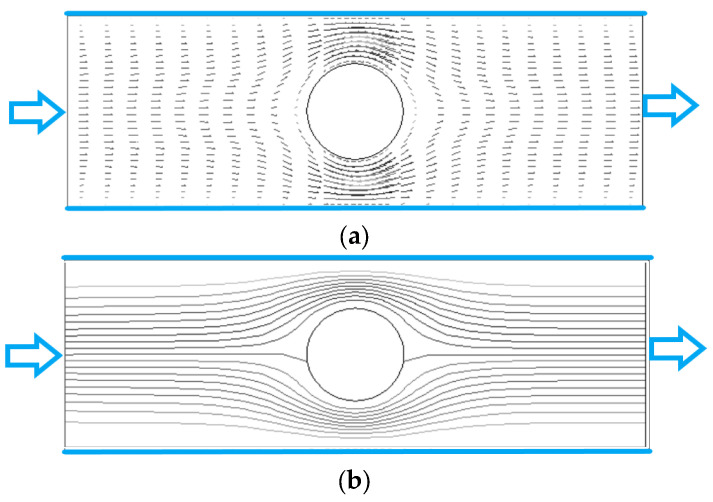
Fluid velocity vector and streamline of flow (Re = 2, *n* = 0.7). (**a**) velocity vector. (**b**) streamline.

**Figure 4:**
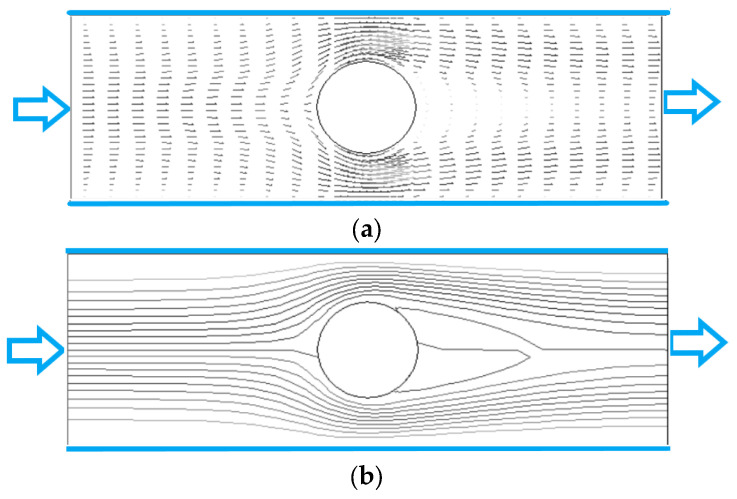
Fluid velocity vector and streamline of flow (Re = 200, *n* = 0.7). (**a**) velocity vector. (**b**) streamline.

Figure 3 and Figure 4 show the fluid velocity vector and streamline distribution of the flow for Re = 2, 200 and *n* = 0.7. The Reynolds number is defined as Re=4Ru¯f/ν (4R is the channel width as shown in Figure 2, ${\bar{u}}_{f}$ is the mean velocity, and ν is the viscosity). We can see that when Re is small (Re = 2) as shown in Figure 3, the distributions of velocity vector and streamline are almost symmetrical about the center of the cylinder, which is consistent with the flow characteristics of small Re. When Re is large (Re = 200) as shown in Figure 4, the distributions of velocity vector and streamline in front of the cylinder are similar to those in Figure 3, but the difference is that there is obvious wake in the flow behind the cylinder because there is zero values in the velocity vector distribution in Figure 4a and vortex in the streamline distribution in Figure 4b.

In the unbounded flow around a cylinder of Newtonian fluid, there will be a Karman vortex street behind the cylinder when Re > 40. In Figure 4b, the Karman vortex street does not appear even if Re = 200. The reasons may be attributed to two aspects. One is that the existence of the upper and lower walls inhibits the generation of Karman vortex behind the cylinder because the walls have a large shear force on the fluid, and its direction is opposite to the shear force generated by the cylinder, which reduces the rotation of the flow behind the cylinder. Another reason is that the fluid in Figure 4 is shear-thinning fluid, and the fluid after thinning also reduces the rotation of the flow behind the cylinder.

**Figure 5:**
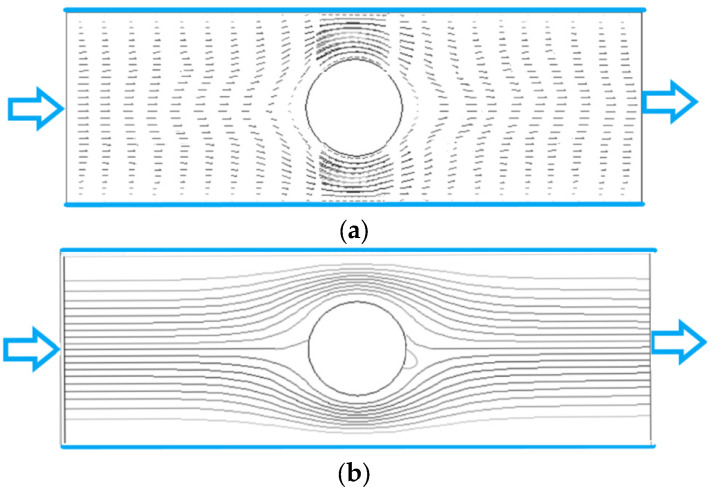
Fluid velocity vector and streamline of flow (Re = 200, *n* = 1.3). (**a**) velocity vector. (**b**) streamline.

Figure 5 shows the fluid velocity vector and streamline distribution of the flow for Re = 200 and *n* = 1.3. Compared to the shear-thinning situation in Figure 4b, the wake behind the cylinder becomes smaller. This is attributed to that the increased viscosity results in the reduction of the local Reynolds number.

### 4.2. Effect of Initial Orientation of Particles on the Distribution of Particles

**Figure 6:**
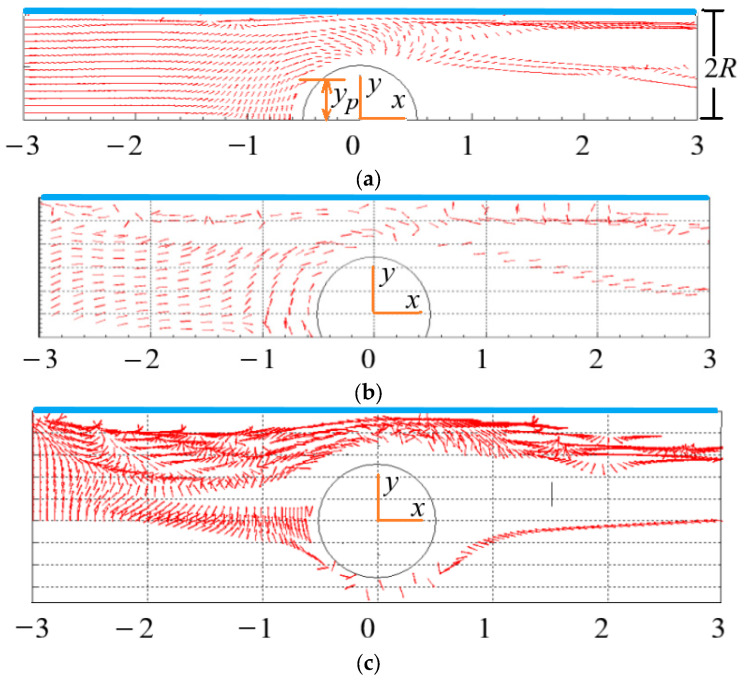
Spatial and orientation distributions of particles with different initial orientation (Re = 2, *n* = 1.3, *β* = 8). (**a**) initial orientation *θ*_0_ = 0°. (**b**) initial orientation *θ*_0_ = 45°. (**c**) initial orientation *θ*_0_ = 90°.

When the rodlike particles flow through the cylinder between two planes, the flow goes through the process of contraction and expansion. For a particle initially located at the inlet, not all particles in the transverse position *y* can bypass the cylinder, and some particles will stay in the detention area in front of the cylinder. As shown in Figure 6a, the transverse position of the inlet where particles can bypass the cylinder is defined as *y_p_* (if transverse position *y*_0_ < *y_p_*, the particles cannot bypass the cylinder), then *y_p_* depends on the initial orientation *θ*_0_ of particles at the inlet.

Figure 6a shows the spatial and orientation distributions of particles with *θ*_0_= 0°, we can see that *y_p_* ≈ 0.3 (2*R*), i.e., the particles with *y*_0_ < 0.3 (2*R*) cannot bypass the cylinder. The spatial and orientation distributions of particles with *θ*_0_ = 45° are shown in Figure 6b where the particles, after entering the flow, first move towards the center, and the orientation angle *θ* decreases. When the particle approaches the cylinder, the values of *θ* increase, and the transverse velocity component of the particle becomes positive and gradually increases. At this time, *y_p_* ≈ 0.25(2*R*), i.e., the particle with *y*_0_ < 0.25 (2*R*) cannot bypass the cylinder. Comparing with Figure 6a, more particles with *θ*_0_ = 45° can bypass the cylinder than the particles with *θ*_0_ = 0°. Figure 6c shows the case for the particles with *θ*_0_ = 90°, it can be seen that the particles, after entering the flow, exhibit obvious rotation, and *y_p_* ≈ 0.15 (2*R*) in this case, indicating more particles can bypass the cylinder compared with the above two situations. Therefore, the initial orientation of particles has a great influence on whether particles can bypass the cylinder.

### 4.3. Effect of Re on the Distribution of Particles

**Figure 7:**
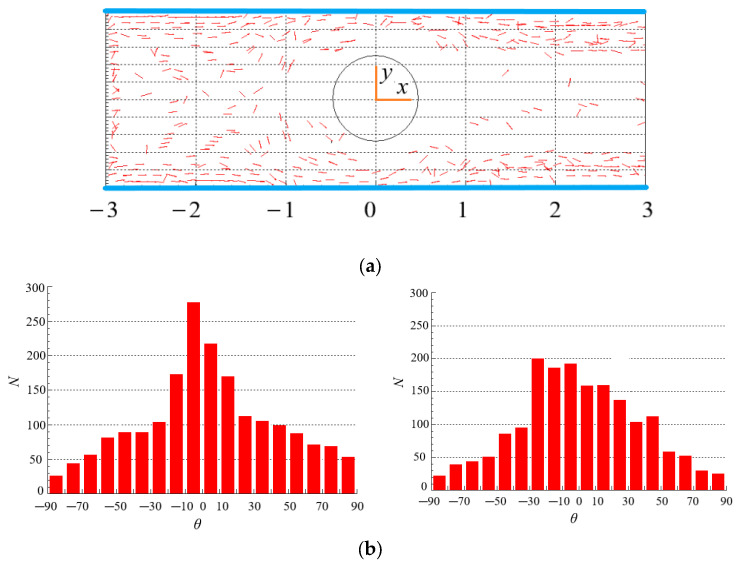
Spatial and orientation distributions of particles at Re = 2 (*n* = 1, *β* = 8). (**a**) spatial distribution. (**b**) orientation distribution: (**left**): contraction flow (*x* < 0); (**right**): expansion flow (*x* > 0).

**Figure 8:**
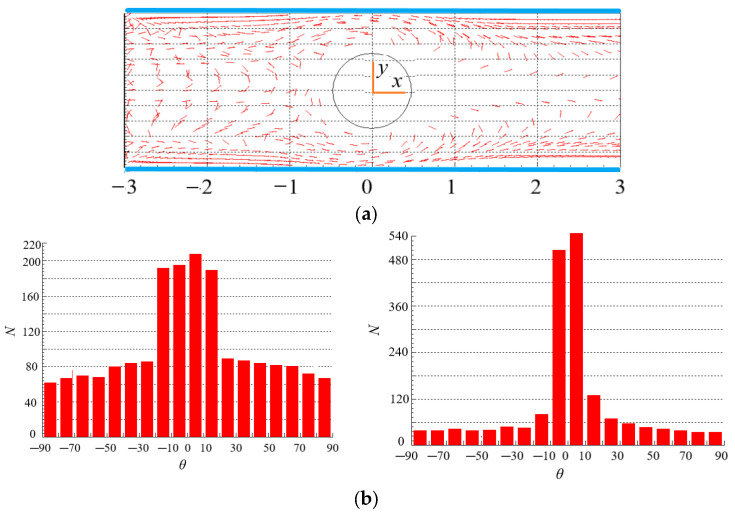
Spatial and orientation distributions of particles at Re = 200 (*n* = 1, *β* = 8). (**a**) spatial distribution. (**b**) orientation distribution: (**left**): contraction flow (*x* < 0); (**right**): expansion flow (*x* > 0).

**Figure 9:**
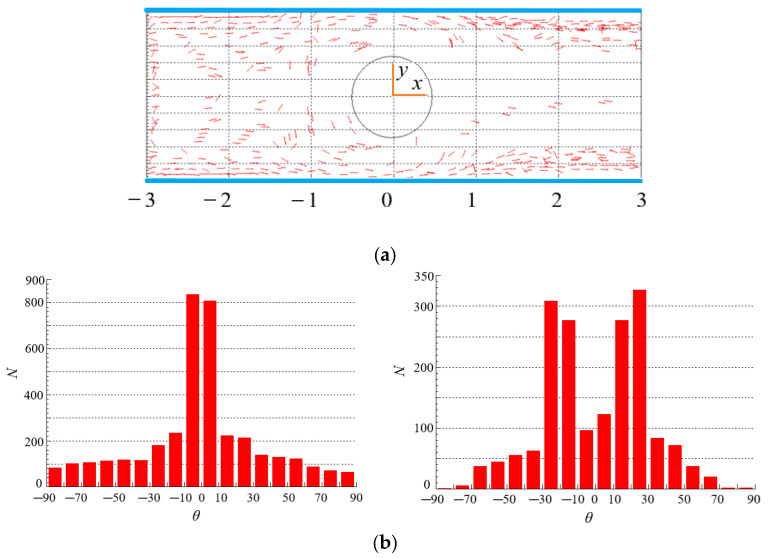
Spatial and orientation distributions of particles at *n* = 1.3 (Re = 200, *β* = 8). (**a**) spatial distribution. (**b**) orientation distribution: (**left**): contraction flow (*x* < 0); (**right**): expansion flow (*x* > 0).

The spatial and orientation distributions of particles for different Re are shown in Figure 7 and Figure 8 where *θ* is the included angle between particle principal axis and horizontal direction, *N* is the number of particles. In Figure 7a and Figure 8a, the number of particles around the cylinder, especially at the tail of the cylinder, is small, i.e., the existence of the cylinder leads to the uneven spatial distribution of particles. The reasons for the phenomenon that only some particles present in the wake are: on the one hand, when particles flow through the cylinder, they are squeezed near the wall, and cannot quickly enter the central region after passing through the cylinder. On the other hand, the rotation of the vortex in the wake of the cylinder throws the particles to the area near the walls. Comparing the two figures, it can be seen that Re has no obvious effect on the spatial distribution of particles. Figure 7a and Figure 8a are the cases for Newtonian fluids (*n* = 1). However, the same trend is observed for non-Newtonian fluids (*n* = 1.3) as shown in Figure 9a (Re = 200) and Section 4.5.

Figure 7b and Figure 8b show the number of particles with different *θ* for different Re. In the contraction flow (*x* < 0), more particles have a small *θ* value, indicating that the orientation of more particles points to the flow direction. However, the contraction flow changes the velocity gradient and shear rate of the flow, so there are many particles with different orientations. Moreover, there is little difference in particle spatial distribution under different Re. In the expansion flow (*x* > 0), the orientation distribution of particles is different with that in the contraction flow, and the difference is more obvious at large Re. The reason is that a wake is formed behind the cylinder when the flow bypasses the cylinder, resulting in changes in the velocity distribution and velocity gradient of the flow that affect the particle orientation. In the case of large Re as shown in Figure 8b, most particles are oriented in the flow direction (small *θ*), which can be attributed to that the wake area behind the cylinder is larger at large Re, while there exist large velocity gradient and strong shear force in wake area. The strong shear makes the torque acting on the particles larger and the rotation of the particles faster. The result of the torque action makes the particle orientation point to the flow direction. In addition, the particles near the wall show obvious orientation along the flow direction under strong shear force in Figure 8b. From this, we can draw the conclusion that Re has a great influence on the orientation distribution of rodlike particles in the contraction and expansion flow.

### 4.4. Effect of n on the Distribution of Particles

**Figure 10:**
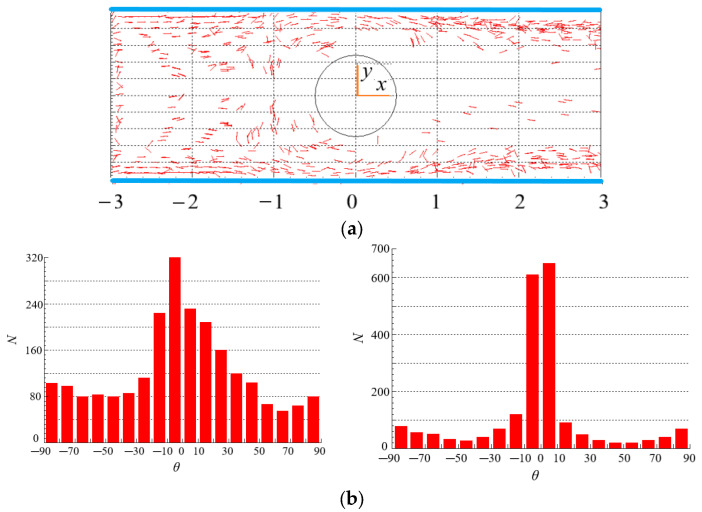
Spatial and orientation distributions of particles at *n* = 0.7 (Re = 200, *β* = 8). (**a**) spatial distribution. (**b**) orientation distribution: (**left**): contraction flow (*x* < 0); (**right**): expansion flow (*x* > 0).

Figure 9 and Figure 10 show the spatial and orientation distributions of particles for different power index *n*. Under the same velocity gradient and fluid shear rate, the force and torque of fluid with high viscosity acting on particles are also large as shown in Equations (1) and (7). The higher the shear-thickening degree, the larger the viscosity. In Figure 9a, particles are dispersed in the whole area in the contraction flow, while in the expansion flow, more particles are gathered near the two walls, and less particles are in the center area, which is caused by the rotation of the vortex in the wake of the cylinder, and the rotation throws the particles to the walls. Comparing Figure 9a and Figure 10a, we can see that the power index *n* has an effect on the spatial distribution of particles. It is worth pointing out that the different streamlines as shown in Figure 4b and Figure 5b do not affect the spatial distribution significantly. The reason may be that although there are different streamlines between the two scenarios, the difference in velocity is not as obvious as the difference in streamline, and the particle motion is mainly influenced by velocity.

As for the orientation distribution of particles, for the case of shear-thickening fluid in Figure 9b, more particles orient to the flow direction in the contraction flow (*x* < 0) because of high viscosity and even large force and torque acting on particles as shown in Equations (13) and (14), while viscosity and force are related to the velocity gradient as shown in Equation (15). By comparison, the orientation distribution of particles is more dispersed in the contraction flow for the shear-thinning fluid as shown in Figure 10b. However, more particles orient to the flow direction in the expansion flow (*x* > 0) for the case of shear-thinning fluid in Figure 10b, while the orientation distribution of particles exhibits two peaks in −30°~−10° and 10°~30° in the expansion flow for the shear-thickening fluid as shown in Figure 9b. The reason is that the velocity distributions of shear-thinning fluid and shear-thickening fluid are different after fluid passing through the cylinder, the velocity distribution of the shear-thinning fluid produces larger shear rate as well as viscosity and force as shown in Equations (13)–(15), so that making more particles orient to the flow direction, but the velocity distribution of the shear-thinning make the orientation distribution exhibit two peaks. As a consequence, the power index *n* has a significant effect on the orientation distribution of particles in the contraction and expansion flow. In shear-thickening fluid, when the flow changes from contraction to expansion, the orientation distribution of particles becomes more dispersed; while in shear-thinning fluid, the opposite is true.

Comparing Figure 9b and Figure 10b, we can see that there are more particles with an orientation of 90° in shear-thinning fluids than that in shear-thickening fluids. This is because the force and torque exerted by the shear-thinning fluid on the particles are relatively small, and the initial orientation of the particles at the inlet is randomly distributed.

### 4.5. Effect of Particle Aspect Ratio on the Distribution of Particles

**Figure 11:**
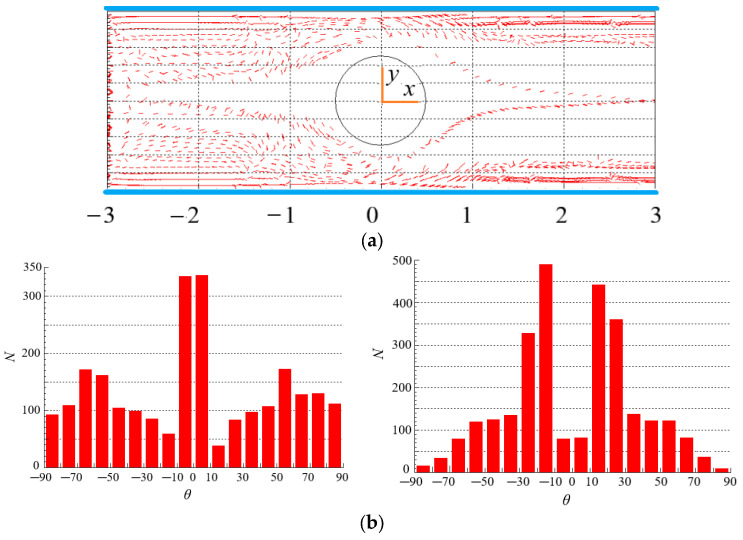
Spatial and orientation distributions of particles at *β* = 4 (Re = 2, *n* = 1.3). (**a**) spatial distribution. (**b**) orientation distribution: (**left**): contraction flow (*x* < 0); (**right**): expansion flow (*x* > 0).

**Figure 12:**
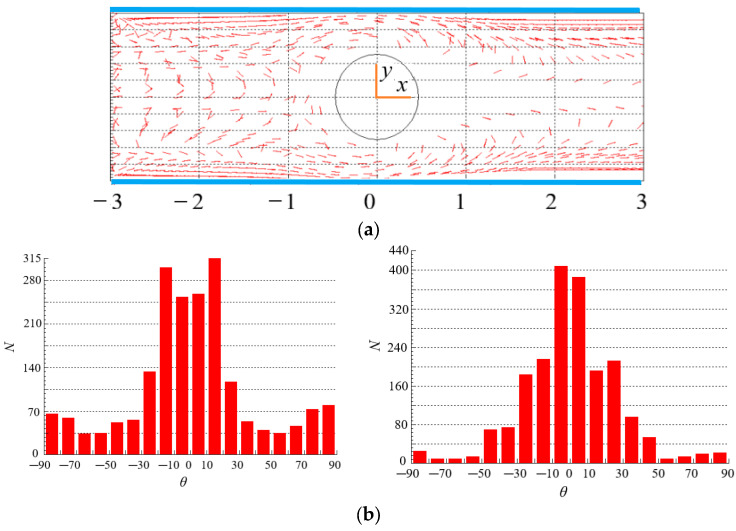
Spatial and orientation distributions of particles at *β* = 8 (Re = 2, *n* = 1.3). (**a**) spatial distribution. (**b**) orientation distribution: (**left**): contraction flow (*x* < 0); (**right**): expansion flow (*x* > 0).

The spatial distribution of particles for *β* = 4 in Figure 11a is more regular compared with the spatial distribution of particles for *β* = 8 in Figure 12a, even there is a symmetric distribution about *x* = 0 in the vicinity of the central line. This is because the particles with small aspect ratio follow the fluid better, and their distribution can better reflect the symmetry characteristics of the flow around the cylinder at Re = 2. Therefore, particle aspect ratio has a significant impact on the spatial distribution of particles in contraction flow and expansion flow.

In Figure 11b, the orientation angles of more particles are at *θ* = −10~10, i.e., the orientation points to the flow direction in the contraction flow. The reason is that, in addition to shearing, there exists a strong extension effect in the contraction flow, which makes the particles align with the flow direction. However, the extension effect does not exist in the expansion flow, the orientation angles of more particles are at *θ* = −10~−30 and 10~30, i.e., the orientation is close to the flow direction. The orientation distributions of particles with large aspect ratio (*β* = 8) are shown in Figure 12b. It can be seen that there are more particles orient to the flow direction in expansion flow than that in contraction flow. Comparing with Figure 11b, more particles point to the flow direction for *β* = 8 than that for *β* = 4. The reason may be that, as shown in Equations (1)–(3) and (7)–(10), the forces and moments acting on particles are directly related to the particle aspect ratio*β*, in particular, the angular acceleration of particles is directly proportional to the*β*, large torque and even large angular acceleration make the orientation of particles easier to point to the flow direction, thus the particles with a large aspect ratio tend to align with the flow direction more obviously. Moreover, particle aspect ratio has a stronger effect on the orientation distribution than spatial distribution by comparing Figure 11 and Figure 12.

## 5. Conclusions

The contraction and expansion flow containing rodlike particles in power-law fluid is studied numerically. The movement, spatial and orientation distribution of particles are described by the equations of force and moment exerted on the particles. The fluid velocity vector and streamline of flow are given at different Reynolds number. The effects of Re, power index *n* and particle aspect ratio *β* on the spatial and orientation distributions of particles are analyzed. The main conclusions can be summarized as follows.

Distributions of velocity vector and streamline are almost symmetrical about the center of the cylinder when Re = 2. When Re = 200, the distributions of velocity vector and streamline in front of the cylinder are similar to those when Re = 2, but the difference is that there is obvious wake in the flow behind the cylinder. There is no Karman vortex street like Newtonian fluid in the power-law fluid even if Re = 200.

Te number of particles around the cylinder, especially at the tail of the cylinder is small. The existence of the cylinder leads to the uneven spatial distribution of particles. For the shear-thickening fluid, particles are dispersed in the whole area in the contraction flow, while more particles are gathered near the two walls in the expansion flow. The spatial distribution of particles with small *β* is more regular. *Β* has a significant, *n* has a moderate, but Re has a small impact on the spatial distribution of particles in the contraction and expansion flow.

The orientation distributions of particles in the contraction flow and expansion flow are different, and the difference degree is more obvious at large Re. In the case of large Re, most particles are oriented in the flow direction. The particles near the wall show obvious orientation along the flow direction. In shear-thickening fluid, when the flow changes from contraction to expansion, the orientation distribution of particles becomes more dispersed; while in shear-thinning fluid, the opposite is true. The orientation of more particles points to the flow direction in the contraction flow, while is close to the direction of flow in the expansion flow. More particles orient to the flow direction in expansion flow than that in contraction flow. The particles with a large *β* tend to align with the flow direction more obviously. Re, *n* and *β* have great influence on the orientation distribution of particles in the contraction and expansion flow.

Whether the particles initially located at the inlet can bypass the cylinder depends on the transverse position and initial orientation of the particles at the inlet. The number of particles with *θ*_0_ = 90° bypassing the cylinder is the largest, followed by *θ*_0_ = 45° and *θ*_0_ = 0°. The conclusions obtained in this paper have reference value for practical engineering applications.
